# Retrospective Clinical Evaluation of RMGIC/GIC Class V Restorations

**DOI:** 10.3390/dj11090225

**Published:** 2023-09-20

**Authors:** Maria Jacinta M. C. Santos, Lucy Leon, Imad Siddique, Sheila Butler

**Affiliations:** 1 Division of Restorative Dentistry, Schulich School of Medicine and Dentistry, Western University, London, ON N6A 5C1, Canada; sheila.butler@schulich.uwo.ca; 2 Schulich School of Medicine and Dentistry, Western University, London, ON N6A 5C1, Canada; lleon2020@dents.uwo.ca (L.L.); isiddique2019@dents.uwo.ca (I.S.)

**Keywords:** glass ionomer, resin modified glass ionomer, Class V, clinical evaluation

## Abstract

The aim of this retrospective study was to evaluate the clinical performance of glass-ionomer cement (GIC) and resin-modified glass-ionomer cement (RMGIC) materials in Class V carious cervical lesions restored by dental students. Ninety-six (96) restorations performed with either GIC (Fuji IX) (n = 39) or RMGIC (Fuji II LC) (n = 57) were evaluated using the modified USPHS criteria by two independent investigators at two follow-up evaluations (two years apart). The Fisher statistical test was used to compare USPHS criteria and examine significant differences, with a significance level set at *p* < 0.05. The Kaplan-Meier algorithm was used to calculate the survival probability. The overall success rate of Class V restorations was 72.9% at the second follow-up evaluation, with restorations ranging in age from 2.5 to 3.5 years. The RMGIC (Fuji II LC) restorations exhibited a significantly higher overall success rate compared to the GIC (Fuji IX) restorations (*p* = 0.0104). Significant differences were observed in retention (*p* = 0.0034) and color match (*p* = 0.0023).

## 1. Introduction

Carious and non-carious cervical lesions are common clinical findings. The major drawbacks of these lesions are related to the limitted or absent enamel present at the cervical margin, and proximity to the gingiva, which may result in contamination with crevicular fluid and/or blood during restorative procedures. Glass ionomer cement (GIC) and resin-modified glass-ionomer cement (RMGIC) materials are commonly used to restore Class V carious and non-carious lesions. Systematic reviews have shown a high retention rate of resin-based glass ionomer materials in non-carious cervical lesions compared to resin composite restorations [1,2]. However, a smaller number of studies have been conducted to compare the performance of these materials in Class V carious lesions.

GICs are acid-based cements that result from the reaction between an ion-leachable fluoro-aluminosilicate glass powder and an aqueous solution of polyalkenoic acids [3]. Despite their ability to bond chemically and micro-mechanically to dental structures, GICs have certain limitations when compared to resin composite materials. These limitations include poor handling, limited esthetic results, sensitivity to moisture during initial setting, and inferior physical-mechanical properties [4]. To enhance the properties of GICs, a water-soluble resin monomer called 2-hydroxyethylmethacrylate (HEMA) has been added to their formulation [5], resulting in an improved material. RMGICs are polymerized by a light source in addition to the acid-based reaction inherent in the glass ionomers’ setting mechanism. Compared to conventional GICs, RMGICs exhibit increased resistance to microleakage, stronger bonding to tooth structure, and lower solubility [6]. Furthermore, they are less prone to crack formation and demonstrate superior plastic deformation when compared to GICs [7].

One of the significant advantages offered by these materials is their fluoride-releasing ability. Previous studies [8,9,10] have highlighted the benefits of GIC materials due to their hydrophilic properties and their capacity to act as bioactive substances when the oral pH changes, facilitating potential ion exchange within the tooth structure. GICs and RMGICs initially release fluoride rapidly, followed by a sustained release at a lower level that can be maintained in the long term. This is attributed to the reservoir effect, where the release and uptake of fluoride ions depend on the fluoride concentration in the oral environment [11]. However, some previous studies have raised concerns about the effectiveness of GICs and RMGICs in inhibiting demineralization and promoting enamel remineralization in clinical settings [12,13,14,15].

The rationale for conducting this study was based on the observation of inconsistency among clinical instructors when indicating the use of GI or RMGI materials to restore Class V carious lesions in the clinical setting. The authors are professors in the field of Restorative Dentistry and have consistently witnessed variations in personal preferences among the indications for these two materials. While there is an extensive body of scientific literature concerning these materials in Non-Carious Cervical Lesions (NCCL), the volume of research pertaining to cervical carious lesions remains comparatively small. To better understand the clinical behavior of these two restorative materials in Class V carious lesions, the objective of this investigation was to assess the performance of GIC and RMGIC restorations placed by dental students in the clinical setting. The null hypothesis being tested was that there would be no difference in the clinical performance of GIC and RMGIC restorations between the first and second follow-up evaluations, which were conducted two years apart.

## 2. Materials and Methods

This study was conducted in accordance with research guidelines involving human subjects and received independent review and approval from the Research Ethics Board of the Western University Health Science Research Ethics Board (H.S.R.E.B.) in Canada (Approval #107864). Prior to the clinical evaluation of each patient, a comprehensive understanding of the study was provided, and written informed consent was obtained.

Class V carious lesions restored by predoctoral students at the main Adult Dental Clinic of Schulich Medicine & Dentistry—Western University were identified using the school billing system. Patients who had undergone Class V restorations using type II restorative cements, specifically resin-modified glass-ionomer cement (RMGIC) (Fuji II LC, GC America Inc., Alsip, IL, USA) or glass-ionomer cement (GIC) (Fuji IX, GC America Inc., Alsip, IL, USA), were identified from the patients’ records. These patients were contacted by a dental student and invited to voluntarily participate in this retrospective study at two different follow-up points. Class V carious lesions restored with resin composite and amalgam, as well as Class V non-carious lesions, were excluded from this analysis. The Class V preparations and restorations were carried out by third and fourth-year predoctoral students, under the supervision of a full-time faculty member in the Department of Restorative Dentistry or a part-time faculty instructor.

The treatments performed and information regarding the treatment protocol were documented in the patients’ records. In cases where no detailed information was available in the patients’ dental treatment records, the patients’ data were not included in the research. The teeth designated for restoration were isolated using cotton rolls and retraction cords. Carious lesions were removed utilizing spoon excavators and slow-speed round carbide burs appropriate for the cavity’s size. To shape the final contour of the preparation, high-speed carbide burs (#330/#245 Brasseler) were employed with continuous water cooling. A 20% polyacrylic acid solution (GC conditioner, GC America Inc., Alsip, IL, USA) was applied to clean the cavity preparations and aid in the release of Ca++ ions to enhance chemical bonding [16,17]. The materials and operative procedures were obtained by cross-referencing them with the patient treatment records.

Both GIC (GC Fuji IX) and RMGIC (GC Fuji II LC) were available in premeasured capsules to facilitate handling and optimize the powder-liquid ratio. The capsules were activated in the capsule applier, mixed for 10 s in an amalgamator, and then directly placed into the cavity preparation. When using RMGIC, the material was light-cured with a LED light curing unit (Bluephase style, Ivoclar Vivadent, Schaan, Liechtenstein) for 20 s, according to the manufacturer’s instructions. Following the completion of the setting process, restorations were contoured using finishing diamond burs and polished using rubber cups and points (Cosmedent, Chicago, IL, USA). GC Fuji Varnish (GC America Inc., Alsip, IL, USA) was applied to protect the restorative material against dehydration or water absorption from the oral environment. All clinical procedures were meticulously documented in the patients’ charts. Maintaining uniform procedures among students was possible because they used identical armamentarium. Additionally, over a two-year period in the simulation clinic, they employed either the #330 or #245 bur to prepare Class V lesions. They followed this by mixing the capsulated ionomer materials for 10 s using an amalgamator. Furthermore, during clinic sessions, students were provided with a single container housing the GC conditioner, GIC or RMGI, and Varnish protector to perform ionomer-based restorations. The composition of the restorative materials is outlined in Table 1.

### Data Collection

Patients who had received Class V restorations with GIC (Fuji IX) or RMGIC (Fuji II LC) at the Schulich Dental Clinic were contacted via telephone and invited to participate in two follow-up visits. The second follow-up evaluation was scheduled two years after the initial evaluation. During these retrospective evaluations, the age of the restorations ranged from 6 months to 1.5 years during the first follow-up, and from 2.5 years to 3.5 years during the second follow-up. The patients were provided with information about the research methodology, the potential risks and benefits of their participation, and written informed consents were obtained. The restorations were evaluated using the United States Public Health Service (USPHS) criteria [18], slightly modified by van Dijken [19] as outlined in Table 2. Two independent calibrated investigators assessed the restorations. The investigators worked as a team but independently evaluated the restorations using mirrors and dental explorers. In case of disagreements, a joint examination was conducted to reach a consensus on the final score for each evaluated aspect of the restorations. Interexaminer reliability (Kappa) exceeded 0.85 for all criteria, indicating a strong level of agreement among examiners. Intra reliability was not determined because both evaluators possessed prior experience with the modified USPHS criteria. Patients were asked about their satisfaction with the restoration and whether they experienced any sensitivity or discomfort following the placement. The type of ionomer-based restorative material used (GIC/RMGIC) and the teeth that were restored were recorded. Clinical photographs were taken for selected patients to illustrate specific aspects of the criteria.

During the evaluation process, Class V restorations were assessed using a modified USPHS criteria that encompassed seven different aspects. Failures were defined as restorations that were lost or fractured prior to the evaluation or those requiring replacement. To calculate survival probability, the Kaplan-Meier algorithm was employed using GraphPad Prism 6. Statistical analyses were conducted utilizing Fisher and McNemar tests with a significance level of 0.05. The Fisher test was utilized to compare the aspects evaluated in the USPHS criteria and identify any significant differences. On the other hand, the McNemar test was employed to compare data from the two evaluation periods for each restorative material.

## 3. Results

A total of 96 restorations were evaluated in 36 patients, resulting in a recall rate of 100%. Among these, 39 restorations were performed using GIC (Fuji IX) and 57 restorations were performed using RMGIC (Fuji II LC). Of the 36 assessed patients, 22 were female and 14 were male, with an average age of 67 years.

Table 3 and Table 4 present the results of the clinical evaluation of Class V restorations using GIC (Fuji IX) and RMGIC (Fuji II LC), respectively. The McNemar statistical test revealed a significant difference in color match between GIC (Fuji IX) and RMGIC (Fuji II LC) restorations (*p* = 0.0023) during the second follow-up evaluation.

**Figure 1:**
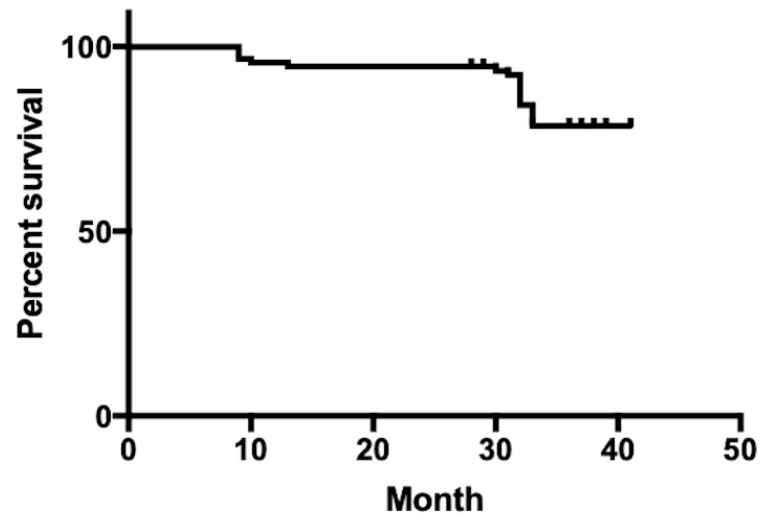
Retention of GIC and RMGIC Class V restorations based on Kaplan Meier survival analysis.

According to the Kaplan-Meier methodology, the estimated retention of all Class V restorations was 94.6% at 12 months and 72.9% at 40 months, as shown in Figure 1. The retention of RMGIC (Fuji II LC) restorations was significantly higher than that of GIC (Fuji IX) restorations (*p* = 0.0034). The loss rate for GIC (Fuji IX) Class V restorations was 17.9%, while for RMGIC (Fuji II LC) Class V restorations, it was 1.8%.

Out of the 96 restorations assessed, a total of 26 failed between the first and second follow-up evaluations, resulting in an overall failure rate of 27.1%. GIC (Fuji IX) restorations exhibited a significantly higher failure rate in comparison to RMGIC (*p* = 0.0104).

**Figure 2:**
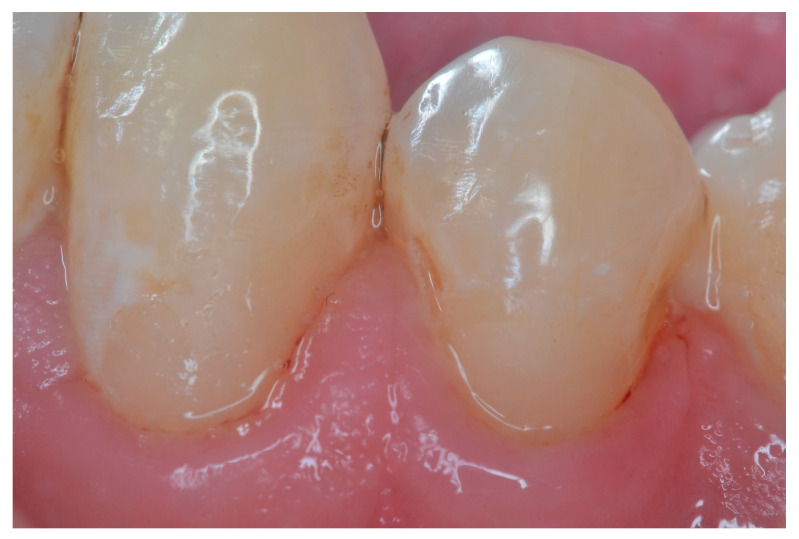
Class V RMGIC restorations on the lower left canine and premolar—2nd follow-up evaluation. Most aspects of the modified criteria were rated as Alfa. The surface texture of the lower left canine was rated as Bravo.

**Figure 3:**
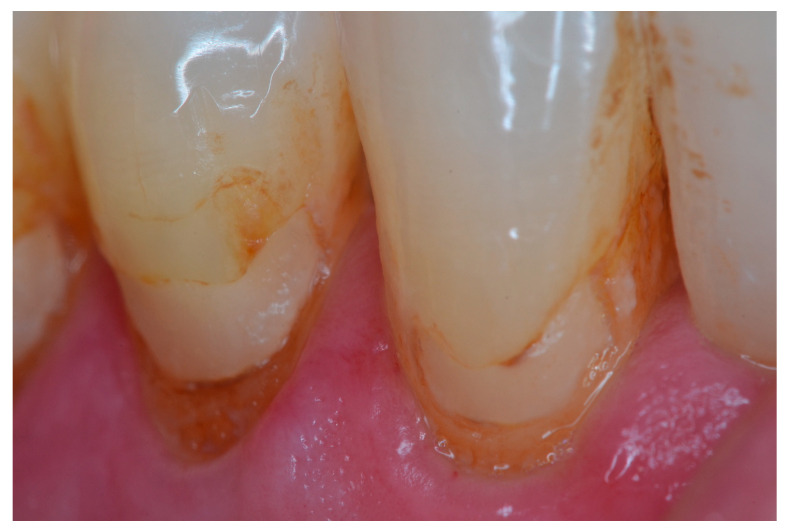
Class V RMGIC restorations on the lower right canine and premolar—2nd follow-up evaluation. Bravo rating for marginal discoloration, surface roughness, anatomy, and color match. Charlie rating for marginal integrity.

**Figure 4:**
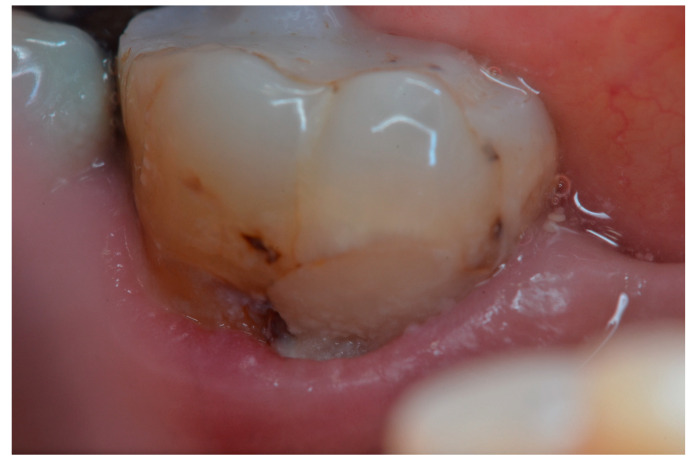
Class V GIC restorations on the 1st lower left molar—2nd follow-up evaluation, showing the presence of secondary caries (Bravo). Bravo rating for marginal discoloration, surface roughness and anatomy. Charlie rating for color match, and Delta rating for marginal integrity.

**Figure 5:**
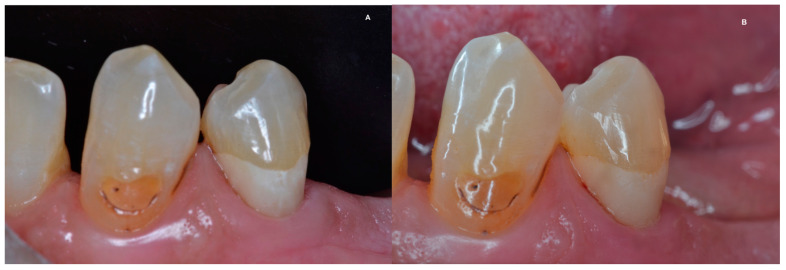
(**A**,**B**) Class V GIC restoration on lower left first premolar at the 1st and 2nd follow-up evaluations. Alfa ratings for most aspects, but Bravo rating for surface texture and Charlie rating for color match.

Figure 2, Figure 3, Figure 4 and Figure 5 illustrate clinical results of GIC and RMGIC Class V restorations evaluated in this study.

## 4. Discussion

In the present study, the clinical performance of GIC (Fuji IX) and RMGIC (Fuji II LC) Class V restorations showed a decline over the 3.5-year study period. Therefore, the null hypothesis, which proposed no difference in the clinical performance between GIC and RMGIC restorations at the 1st and 2nd follow-up evaluations, was rejected.

Folwaczny et al. [20] reported a 6% loss of Fuji II LC cervical restorations in carious lesions after a two-year evaluation. Stewardson et al. [21] assessed different restorative materials in Class V lesions and reported a significantly higher survival rate for RMGIC Class V restorations (78.6%) compared to GIC Class V restorations (50.6%) in carious lesions after five years, which aligns with the findings of this investigation. Furthermore, a systematic review by Hayes et al. [22] indicated that the 12-month survival rate of RMGIC Class V restorations was 83.0%, while it was only 63.8% for GIC restorations. The higher retention of RMGI compared to GIC has been attributed to its unique bonding mechanism, which combines the advantages of glass ionomer cements and resin-based materials. The glass ionomer component forms a chemical bond with the tooth structure, while the resin component provides a mechanical bond [23]. Therefore, the bonding mechanisms of RMGI to the tooth tissue are twofold: micromechanical interlocking and chemical interaction between the carboxyl groups of the ionomer materials and the calcium of the dental structure [23]. Moreover, the superior clinical performance of RMGICs has been ascribed to the improved mechanical properties of RMGIC compared to GIC [4,21,24,25,26].

Long-term retention of dental restorations is a crucial requirement for any dental restorative material, and it can be influenced by various factors. Within the oral environment, restorative materials are continuously exposed to mechanical and chemical degradation. Various factors, including moisture, physical and mechanical stresses, parafunctional habits, diet, pH fluctuations, and temperature changes, can contribute to the deterioration of the tooth-restoration interface [27]. Furthermore, the patient’s age, operator’s skill, cavity preparation, and choice of restorative material all had an impact on the survival of cervical restorations [20,28].

To enhance the longevity of restorations, the use of surface-modifying agents has been proposed to improve the bond strength with the dental structures. Among these agents, polyacrylic acid (PAA) is commonly utilized as a conditioner before bonding with glass ionomer materials due to its high molecular weight and ability to clean the dentin surface without fully unplugging the dentinal tubules [23,28]. Several studies have reported a significant improvement in the bond strength of Fuji II LC after conditioning with polyacrylic acid [16,29]. This increase in bond strength has been attributed to the cleansing effect of polyacrylic acid, which aids in the removal of debris generated during cavity preparation and induces partial demineralization. This process contributes to the formation of a shallow hybrid layer with a micro-porous, hydroxyapatite-coated collagen network, resembling a self-etching approach [30]. However, several investigations have shown no significant differences in the retention rate of restorations with or without the use of polyacrylic acid [31,32,33]. For instance, van Dijken et al. [32] compared the effect of using a cavity conditioner and water rinsing before restoration placement and found no difference in cumulative loss rate over a 4-year follow-up. Another study compared lesions pretreated with either polyacrylic acid or a water-pumice slurry and reported no significant difference between the two groups [33]. The results published in the scientific literature still present conflicting views regarding the efficacy of using an acid conditioner. In the present study, the polyacrylic acid was used to clean the cavity preparations prior to the placement of the restorative materials due to its ability to cleansing the dentin surface without completely unplugging the dentinal tubules [17,23].

Marginal adaptation and marginal discoloration are important factors that influence the long-term success of restorations and can serve as indicators of potential microleakage [34]. In the present study, no significant differences were found in the marginal adaptation of GIC and RMGIC restorations during the 1st and 2nd follow-up evaluations. However, there was a noticeable increase in the number of Bravo ratings at the 2nd follow-up visit for both GIC (Fuji IX) and RMGIC (Fuji II LC) restorations. Another study observed a decrease in the number of Alfa ratings for marginal discoloration after 18 months of evaluation, particularly at the cervical margins of the lesions [35].

An incremental decrease in marginal integrity was also observed between the first and second follow-up evaluations. Folwaczny et al. [36] reported that RMGICs exhibited poorer marginal integrity compared to resin-composite restorations, with decreased Alfa ratings for marginal discoloration (55% Alfa) after three years. The observed decline in marginal adaptation, as noted in both the present study and previous studies, can be attributed to the challenges associated with handling these highly viscous materials [30]. GIC and RMGIC materials have a sticky and viscous nature, which can hinder their easy placement and contouring during cavity preparation, in addition to their limited working time. Consequently, this can lead to suboptimal adaptation of the restoration to the tooth structure. Furthermore, a previous study highlighted the influence of the materials’ microstructure on the wear rate and emphasized that the wear of glass ionomer materials is affected by factors such as the size and shape of the inorganic particles and the continuity of the matrix/glass particle interface [37].

Secondary caries was responsible for 10.2% of failures in GIC restorations and 12.2% of failures in RMGI restorations. There are in-vitro claims in the scientific literature regarding the anti-cariogenic property of glass ionomer materials, which is attributed to their long-term fluoride-releasing capability [38]. GIC and RMGIC materials have the ability to replenish themselves with fluoride from the surrounding environment, depending on the concentration gradient. Studies have reported that regular use of fluoride toothpaste can provide sufficient fluoride to be absorbed into the glass ionomer, which can then be released into the adjacent tooth structure [17,30]. Tyas et al. [34] reported a 1% occurrence of secondary carious lesions after a five-year evaluation period and suggested that GIC presents less potential for recurrent caries compared to resin composite. However, a recent comprehensive analysis indicated that there was no significant difference in caries occurrence at the margins of GIC restorations when compared to resin composite restorations after a duration of 5 years in clinical practice [39]. Moreover, several studies have questioned the effectiveness of fluoride-releasing materials in caries control, as their capacity to prevent caries has not been clinically proven [12,13,14,15]. Although, in the present study, the highest percentage of bravo scores were observed for surface roughness, marginal integrity and marginal discoloration; secondary caries was the primary reason for restoration failure. However, it is important to highlight that cases with observed secondary caries often exhibited a high degree of plaque accumulation and poor oral hygiene. Several other studies have found that despite the presumed cariostatic effects of glass ionomer materials, secondary caries remains the major cause of restoration failure [40,41]. It is possible that the fluoride released from glass ionomer materials is not sufficient to inhibit bacterial growth associated with secondary caries [12,14]. A recent systematic review and meta-analysis found no significant difference in the occurrence of secondary caries between resin composite and GIC materials [15]. However, further research is needed to gain more clarity on this matter.

In the present study, RMGIC restorations exhibited a significantly higher proportion of Alfa color match ratings compared to GIC restorations (*p* = 0.0023). The inferior color match results observed with the GIC material may be attributed to the extended acid-based polymerization reaction, leading to increased sensitivity to water sorption and dehydration. While GIC (Fuji IX) undergoes a chemical acid-base reaction, RMGIC (Fuji II LC) engages in a dual reaction mechanism, consisting of a rapid polymerization reaction of the resinous component and a slower acid-base reaction. RMGIC materials are considered to be more resistant and less susceptible to moisture due to the presence of resin monomers in their composition, enabling them to set upon light activation [28]. Abdalla et al. [42] evaluated the effectiveness of Fuji II LC in Class V carious cavities and also attributed Bravo scores to color match, anatomic form, cavosurface marginal discoloration, and marginal integrity.

Although GIC and RMGIC restorations can provide satisfactory esthetic results, they are generally not considered ideal materials for use in highly esthetic areas. This is due to their increased roughness and opacity when compared to resin composite materials [34]. In a study by Folwaczny et al. [36], different restorative materials were evaluated in cervical lesions, and it was observed that glass ionomer-based materials often exhibited clinically detectable roughness. In the present investigation, surface roughness presented a high incidence of Bravo rating in both GIC and RMGIC at the first and second follow-up visits. Although Bravo in this category is considered clinically acceptable, these restorative materials did not exhibit an ideal surface texture comparable to the enamel structure. This finding can be explained by the heterogeneous nature of these materials, in which large filler particles are embedded in soft matrices, which contributes to a rougher texture compared to resin composite materials [43]. Additionally, the lack of strong coherence between the polyalkenoate and poly-HEMA matrices and the glass particles can lead to the dislodgement of the glass fillers, resulting in a rough surface texture [44]. Moreover, The dislodgement of filler particles due to wear favours plaque accumulation, resulting in lower Alfa ratings for both surface roughness and color match [20,34].

Achieving the desired anatomical contour with glass ionomer materials can be challenging due to the viscoelastic nature of the materials and their limited working time [30]. At the second follow-up evaluation, an increased number of Bravo ratings were observed for both GIC and RMGIC restorations. These findings are consistent with previous reports, which observed high Bravo ratings for anatomic form [36,44]. Furthermore, GIC and RMGIC materials are more prone to wear, fracture, and chipping, due to their lower strength compared to other restorative materials such as composite resins [39]. This can lead to a compromised anatomical shape over time.

In the present study, the use of GIC and RMGIC materials in capsule form allowed for a more favorable powder/liquid ratio compared to hand-mixed materials [45,46,47]. This optimized ratio contributes to improved mechanical properties and facilitates adequate moisture for effective bonding to the substrate [48]. However, it is important to acknowledge the limitations of retrospective studies, such as the more limited control over treatment protocols compared to prospective studies. Therefore, it is crucial to interpret the results with caution and take into account these limitations when drawing conclusions. On the other hand, findings from retrospective studies may have more practical relevance in real-world clinical settings, where a diverse range of operators work with the restorative materials in various clinical scenarios.

## 5. Conclusions

Within the limitations of this investigation, the following conclusions can be drawn:

- The overall success rate of Class V restorations in this study was 72.9% for restorations ranging from 2.5 to 3.5 years old.
- RMGIC (Fuji II LC) restorations exhibited higher retention rates and better color match compared to GIC (Fuji IX) restorations.

## Figures and Tables

**Table 1:** List of the main restorative materials used in this study.

Material	Brand	Composition	Manufacturer
GIC	Fuji IX Capsule	Fluoroaluminium silicate glass, polyacrylic acid, polybasic carboxylic acid	GC America Inc., Alsip, IL, USA
RMGIC	Fuji II LC Capsule	2-Hydroxyethyl methacrylate, polyacrylic acid, water; 58 wt% fluoroaluminumsilicate	GC America Inc., Alsip, IL, USA
Conditioner	GC Conditioner	20% polyacrylic acid and 3% aluminum chloride	GC America Inc., Alsip, IL, USA

**Table 2:** Modified USPHS criteria used for the clinical evaluation of Class V restorations.

Characteristic	Rating	Criteria
Postoperative sensitivity	Alpha	No postoperative sensitivity
	Bravo	Postoperative sensitivity
Secondary caries	Alpha	No evidence of caries contiguous with margin of restoration
	Bravo	Caries evident contiguous with margin of restoration
Anatomic form	Alpha	Restoration continuous with existing anatomic form
	Bravo	Restorations continuous with existing anatomic form, but not exposing cement material or dentin
	Charlie	Sufficient material lost to expose cement material or dentin
Color match	Alpha	No mismatch in color, shade, and translucency between restoration and adjacent tooth structure
	Bravo	Mismatch between restoration and tooth structure within normal range of color, shade, and translucency
	Charlie	Mismatch between restoration and tooth structure outside normal range of color, shade, and translucency
Surface roughness	Alpha	Smooth surface
	Bravo	Slightly rough or pitted, can be refinished
	Charlie	Rough, cannot be refinished
Marginal discoloration	Alpha	No discoloration on margin between restoration and tooth structure
	Bravo	Discoloration on margin between restoration and tooth structure
	Charlie	Discoloration has penetrated along margin of restorative material in pulpal direction
Marginal integrity	Alpha	No visible evidence of ditching along margin
	Bravo	Visible evidence of ditching along margin not extending to DE junction
	Charlie	Dentin or base is exposed along margin
	Delta	Restoration is mobile, fractured, or missing

**Table 3:** Results of GIC Class V restorations at the 1st and 2nd follow-up evaluations.

Aspects ← Evaluated	GIC ↓ Restorations (Age)	Alfa (%)		Bravo (%)		Charlie (%)		Delta (%)	
		6 Months to 1.5 Years	2.5 Years to 3.5 Years	6 Months to 1.5 Years	2.5 Years to 3.5 Years	6 Months to 1.5 Years	2.5 Years to 3.5 Years	6 Months to 1.5 Years	2.5 Years to 3.5 Years
Postoperative sensitivity		100	100	-	-	-	-	-	-
Secondary caries		96.3	93.5	3.7	6.5	-	-	-	-
Anatomic form		82.9	76.9	14.3	19.2	2.6	3.8	-	-
Color match		51.4	38.5	45.7	61.5	2.9	-	-	-
Surface roughness		1.5	1.3	98.5	98.7	-	-	-	-
Marginal discoloration		77.1	55.8	22.9	44.2	-	-	-	-
Marginal integrity		65.7	29.2	25.7	47.8	5.7	19.2	2.9	3.8

**Table 4:** Results of RMGIC Class V restorations at the 1st and 2nd follow-up evaluations.

Aspects ← Evaluated	RMGIC ↓ Restorations (Age)	Alfa (%)		Bravo (%)		Charlie (%)		Delta (%)	
		6 Months to 1.5 Years	2.5 Years to 3.5 Years	6 Months to 1.5 Years	2.5 Years to 3.5 Years	6 Months to 1.5 Years	2.5 Years to 3.5 Years	6 Months to 1.5 Years	2.5 Years to 3.5 Years
Postoperative sensitivity		98.2	100	1.8	-	-	-	-	-
Secondary caries		98.3	92.3	1.7	7.7				
Anatomic form		94.7	82.7	5.3	17.3	-	-	-	-
Color match		73.7	73.1	22.8	23.0	3.5	3.9	-	-
Surface roughness		1.8	1.6	98.2	96.5	-	1.9	-	-
Marginal discoloration		77.2	57.7	22.8	42.3	-	-	-	-
Marginal integrity		54.4	28.9	43.8	62.0	1.8	9.1	-	-

## Data Availability

Data available on reasonable request to the corresponding author.

## References

[1] Peumans M., Kanumilli P., De Munck J., Van Landuyt K., Lambrechts P., Van Meerbeek B. (2005). Clinical effectiveness of contemporary adhesives: A systematic review of current clinical trials. Dent. Mater..

[2] Santos M.J., Ari N., Steele S., Costella J., Banting D. (2014). Retention of tooth-colored restorations in non-carious cervical lesions—A systematic review. Clin. Oral Investig..

[3] Wilson A.D., Kent B.E. (1972). A new translucent cement for dentistry. The glass ionomer cement. Br. Dent. J..

[4] Xie D., Brantley W.A., Culbertson B.M., Wang G. (2000). Mechanical properties and microstructures of glass-ionomer cements. Dent. Mater..

[5] Wilson A.D. (1989). Developments in glass-ionomer cements. Int. J. Prosthodont..

[6] Ching H.S., Luddin N., Kannan T.P., Ab Rahman I., Abdul Ghani N.R.N. (2018). Modification of glass ionomer cements on their physical-mechanical and antimicrobial properties. J. Esthet. Restor. Dent..

[7] Ruengrungsom C., Burrow M.F., Parashos P., Palamara J.E.A. (2021). Comprehensive characterisation of flexural mechanical properties and a new classification for porosity of 11 contemporary ion-leaching dental restorative materials. J. Mech. Behav. Biomed. Mater..

[8] Mustafa H.A., Soares A.P., Paris S., Elhennawy K., Zaslansky P. (2020). The forgotten merits of GIC restorations: A systematic review. Clin. Oral Investig..

[9] Fricker J.P. (2022). Therapeutic properties of glass-ionomer cements: Their application to orthodontic treatment. Aust. Dent. J..

[10] Dezanetti J.M.P., Nascimento B.L., Orsi J.S.R., Souza E.M. (2022). Effectiveness of glass ionomer cements in the restorative treatment of radiation-related caries—A systematic review. Support. Care Cancer.

[11] Burke F.M., Ray N.J., McConnell R.J. (2006). Fluoride-containing restorative materials. Int. Dent. J..

[12] Cury J.A., de Oliveira B.H., dos Santos A.P., Tenuta L.M. (2016). Are fluoride releasing dental materials clinically effective on caries control?. Dent. Mater..

[13] Yoshihara K., Nagaoka N., Maruo Y., Sano H., Yoshida Y., Van Meerbeek B. (2017). Bacterial adhesion not inhibited by ion-releasing bioactive glass filler. Dent. Mater..

[14] Hara A.T., Turssi C.P., Ando M., González-Cabezas C., Zero D.T., Rodrigues A.L., Serra M.C., Cury J.Á. (2006). Influence of fluoride-releasing restorative material on root dentine secondary caries in situ. Caries Res..

[15] Albelasy E.H., Hamama H.H., Chew H.P., Montaser M., Mahmoud S.H. (2022). Secondary caries and marginal adaptation of ion-releasing versus resin composite restorations: A systematic review and meta-analysis of randomized clinical trials. Sci. Rep..

[16] Tanumiharja M., Burrow M.F., Tyas M.J. (2000). Microtensile bond strengths of glass ionomer (polyalkenoate) cements to dentine using four conditioners. J. Dent..

[17] Inoue S., Van Meerbeek B., Abe Y., Yoshida Y., Lambrechts P., Vanherle G., Sano H. (2001). Effect of remaining dentin thickness and the use of conditioner on micro-tensile bond strength of a glass-ionomer adhesive. Dent. Mater..

[18] Cvar J.F., Ryge G. (2005). Reprint of criteria for the clinical evaluation of dental restorative materials 1971. Clin. Oral Investig.

[19] van Dijken J.W. (2003). A 6-year clinical evaluation of class I poly-acid modified resin composite/resin composite laminate restorations cured with a two-step curing technique. Dent. Mater..

[20] Folwaczny M., Loher C., Mehl A., Kunzelmann K.H., Hinkel R. (2000). Tooth-colored filling materials for the restoration of cervical lesions: A 24-month follow-up study. Oper. Dent..

[21] Stewardson D., Creanor S., Thornley P., Bigg T., Bromage C., Browne A., Cottam D., Dalby D., Gilmour J., Horton J. (2012). The survival of Class V restorations in general dental practice: Part 3, five-year survival. Br. Dent. J..

[22] Hayes M., Brady P., Burke F.M., Finbarr P. (2016). Failures rates of Class V restorations in the management of root caries in adults—A systematic review. Gerodontology.

[23] Rai N., Naik R., Gupta R., Shetty S., Singh A. (2017). Evaluating the Effect of Different Conditioning Agents on the Shear Bond Strength of Resin-Modified Glass Ionomers. Contemp. Clin. Dent..

[24] Pereira L.C., Nunes M.C., Dibb R.G., Powers J.M., Roulet J.F., Navarro M.F. (2002). Mechanical properties and bond strength of glass-ionomer cements. J. Adhes. Dent. Spring.

[25] Moberg M., Brewster J., Nicholson J.W., Roberts H. (2019). Physical property investigation of contemporary glass ionomer and resin-modified glass ionomer restorative materials. Clin. Oral Investig..

[26] Rêgo H.M.C., Butler S., Santos M.J.C. (2022). Evaluation of the Mechanical Properties of Three Resin-Modified Glass-Ionomer Materials. BioMed Res. Int..

[27] van Dijken J.W., Pallesen U. (2008). Long-term dentin retention of etch-and-rinse and self-etch adhesives and a resin-modified glass ionomer cement in non-carious cervical lesions. Dent. Mater..

[28] Stewardson D.A., Thornley P., Bigg T., Bromage C., Browne A., Cottam D., Dalby D., Gilmour J., Horton J., Roberts E. (2011). The survival of Class V restorations in general dental practice. Part 2, early failure. Br. Dent. J..

[29] BarHajizadeh H., Ghavamnasiri M., Namazikhah M.S., Majidinia S., Bagheri M. (2009). Effect of different conditioning protocols on the adhesion of a glass ionomer cement to dentin. J. Contemp. Dent. Pract..

[30] Sidhu S.K. (2011). Glass-ionomer cement restorative materials: A sticky subject?. Aust. Dent. J..

[31] Imbery T.A., Namboodiri A., Duncan A., Amos R., Best A.M., Moon P.C. (2013). Evaluating dentin surface treatments for resin-modified glass ionomer restorative materials. Oper. Dent..

[32] van Dijken J.W. (1996). Four-year evaluation of the effect of 10% polyacrylic acid or water rinsing pretreatment on retention of glass polyalkenoate cement. Eur. J. Oral Sci..

[33] Tyas M.J. (1994). The effect of dentine conditioning with polyacrylic acid on the clinical performance of glass ionomer cement—3-year results. Aust. Dent. J..

[34] Boing T.F., de Geus J.L., Wambier L.M., Loguercio A.D., Reis A., Gomes O.M.M. (2018). Are Glass-Ionomer Cement Restorations in Cervical Lesions More Long-Lasting than Resin-based Composite Resins? A Systematic Review and Meta-Analysis. J. Adhes. Dent..

[35] Gladys S., Van Meerbeek B., Lambrechts P., Vanherle G. (1998). Marginal adaptation and retention of a glass-ionomer, resin-modified glass-ionomers and a polyacid-modified resin composite in cervical Class-V lesions. Dent. Mater..

[36] Folwaczny M., Loher C., Mehl A., Kunzelmann K.H., Hickel R. (2001). Class V lesions restored with four different tooth-colored materials—3-year results. Clin. Oral Investig..

[37] Rodrigues D.S., Buciumeanu M., Martinelli A.E., Nascimento R.M., Henriques B., Silva F.S., Souza J.C.M. (2015). Mechanical Strength and Wear of Dental Glass-Ionomer and Resin Composites Affected by Porosity and Chemical Composition. J. Bio Tribocorros.

[38] Krämer N., Schmidt M., Lücker S., Domann E., Frankenberger R. (2018). Glass ionomer cement inhibits secondary caries in an in vitro biofilm model. Clin. Oral Investig..

[39] Heintze S.D., Loguercio A.D., Hanzen T.A., Reis A., Rousson V. (2022). Clinical efficacy of resin-based direct posterior restorations and glass-ionomer restorations—An updated meta-analysis of clinical outcome parameters. Dent. Mater..

[40] Forss H., Widstrom E. (2004). Reasons for restorative therapy and longevity of restorations in adults. Acta Odontol. Scand..

[41] Manhart J., Garcia-Godoy F., Hickel R. (2002). Direct posterior restorations: Clinical results and new developments. Dent. Clin. N. Am..

[42] Abdalla A.I., Alhadainy H.A., García-Godoy F. (1997). Clinical evaluation of glass ionomers and compomers in Class V carious lesions. Am. J. Dent..

[43] Garshasb M., Santos G.C., Rizkalla A.S., Bohay R., Santos M.J. (2017). Effect of Finishing Procedures on the Surface Roughness of Resin-modified Glass-Ionomer Materials. Compend. Contin. Educ. Dent..

[44] Mahn E., Rousson V., Heintze S. (2015). Meta-Analysis of the Influence of Bonding Parameters on the Clinical Outcome of Tooth-colored Cervical Restorations. J. Adhes. Dent..

[45] Sulaiman T.A., Abdulmajeed A.A., Altitinchi A., Ahmed S.N., Donovan T.E. (2018). Effect of resin-modified glass ionomer cement dispensing/mixing methods on mechanical properties. Oper. Dent..

[46] Oliveira G.L., Carvalho C.N., Carvalho E.M., Bauer J., Leal A.M.A. (2019). The Influence of Mixing Methods on the Compressive Strength and Fluoride Release of Conventional and Resin-Modified Glass Ionomer Cements. Int. J. Dent..

[47] Al-Taee L., Deb S., Banerjee A. (2020). An in vitro assessment of the physical properties of manually- mixed and encapsulated glass-ionomer cements. BDJ Open.

[48] Dowling A.H., Fleming G.J. (2008). Is encapsulation of posterior glass-ionomer restoratives the solution to clinically induced variability introduced on mixing?. Dent. Mater..

